# Strain Solitons in an Epitaxially Strained van der Waals-like Material

**DOI:** 10.1021/acs.nanolett.4c00382

**Published:** 2024-03-18

**Authors:** Jason T. Dong, Hadass S. Inbar, Connor P. Dempsey, Aaron N. Engel, Christopher J. Palmstrøm

**Affiliations:** † Materials Department, University of California, Santa Barbara, California 93106, United States; ‡ Deparment of Electrical and Computer Engineering, University of California, Santa Barbara, California 93106, United States

**Keywords:** van der Waals material, scanning tunneling microscopy, bismuth, strain soliton, molecular beam epitaxy

## Abstract

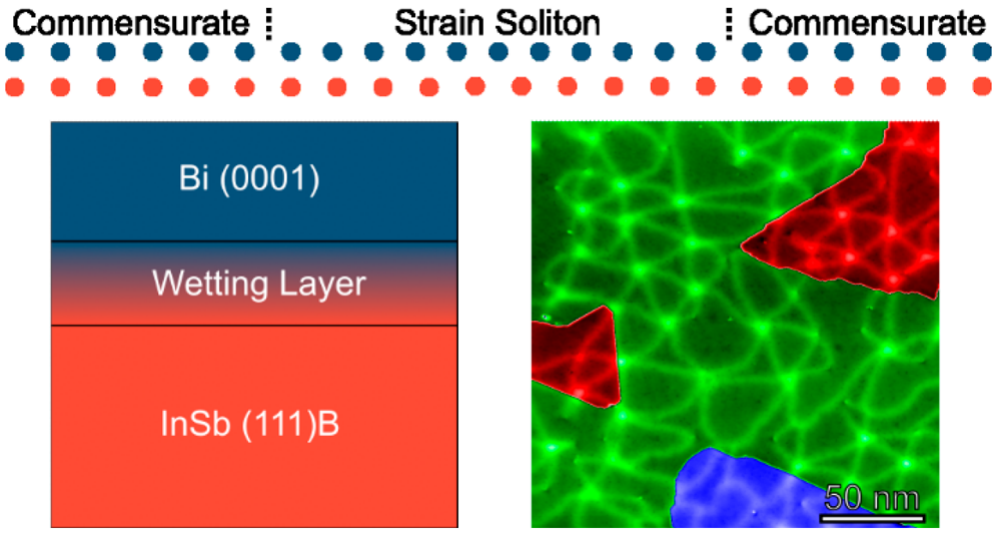

Strain solitons are quasi-dislocations that form in van der Waals materials to relieve the energy associated with lattice or rotational mismatch. Novel electronic properties of strain solitons were predicted and observed. To date, strain solitons have been observed only in exfoliated crystals or mechanically strained crystals. The lack of a scalable approach toward the generation of strain solitons poses a significant challenge in the study of and use of their properties. Here, we report the formation of strain solitons with epitaxial growth of bismuth on InSb(111)B by molecular beam epitaxy. The morphology of the strain solitons for films of varying thickness is characterized with scanning tunneling microscopy, and the local strain state is determined from atomic resolution images. Bending in the solitons is attributed to interactions with the interface, and large angle bending is associated with edge dislocations. Our results enable the scalable generation of strain solitons.

Strain solitons are topological defects in van der Waals materials that act as quasi-dislocations to relieve lattice or rotational mismatch^1^ instead of relaxation by dislocation formation observed in nonlayered materials. The strain solitons are local regions of the crystal where the atoms have rearranged to relieve elastic strain, and the local symmetry is broken. Novel electronic properties such as local band gap modification,^2^ charge carrier confinement,^3^ flatbands,^4−6^ and topological edge modes^7,8^ have been both predicted and observed to occur within the strain solitons. Strain solitons have been experimentally observed in mechanically strained bulk crystals,^3^ exfoliated graphene layers,^1,4,7−10^ hBN multilayers,^11^ and twisted transition metal dichalcogenides.^2,12^ However, a wafer scale approach for the generation of strain solitons has yet to be demonstrated. The lack of a wafer scale method to generate strain solitons poses a significant challenge for the utilization of the novel properties of strain solitons in industrial devices. It is therefore desirable for a wafer scale approach for the formation of strain solitons, such as epitaxial growth, as both a method to investigate the properties of strain solitons and a scalable approach to create functional devices based on strain solitons. Here, to the best of the authors’ knowledge, we are the first to report the formation of strain solitons in bismuth thin films with heteroepitaxial growth on a III–V semiconductor substrate, which enables the scalable creation of strain solitons in a van-der-Waals-like material.

Bismuth is a van-der-Waals-like material (*R*3̅*m*); the crystal structure can be understood as being quasi-hexagonal with buckled layers (one bilayer) of bismuth in a honeycomb lattice (Figure 1A,B). In the bulk, bismuth is a semimetal with large spin–orbit coupling and has demonstrated evidence of a higher order topology.^13^ In this study, strain solitons in bismuth are generated with heteroepitaxial growth of bismuth on InSb (111)B by molecular beam epitaxy. Thicknesses reported in this study are the nominal thickness from the calibrated growth rate. See the Methods section for details of the growth. From the bulk lattice constants and symmetry matching, the InSb (111)B substrate is expected to apply a 0.8% biaxial tensile strain to the bismuth thin film. The layer schematic of the material system is shown in Figure 1C, where the layer structure comprises the InSb (111)B substrate, a bismuth wetting layer, and the bismuth thin film. The first bilayer (BL) of bismuth deposited on InSb forms a wetting layer comprised of atoms arranged in a Sierpiński triangle like pattern^14^ (Figure 1D). Upon subsequent deposition of additional bismuth, islands of (0001) bismuth grow epitaxially on the wetting layer (Figure 1E). While ultrathin bismuth thin films have been observed to grow in the black phosphorus crystal structure,^15^ the quasi-hexagonal bismuth phase is observed to grow in this study for all thicknesses, which likely forms due to the interactions with the substrate. The bismuth islands appear to be oriented in the same direction, which is consistent with the absence of rotational twinning. After additional deposition of bismuth, the islands of bismuth coalesce into a complete, epitaxial single crystal thin film (Figure 1F). See the Supporting Information in S1 for additional XRD of the epitaxial bismuth. Edge states at the terrace edges in our bismuth thin films are observed, these states are not identical to the ones observed in other bismuth thin films.^16^ Further investigations are needed to determine the nature of these states. (see Supporting Information S3).

**Figure 1:**
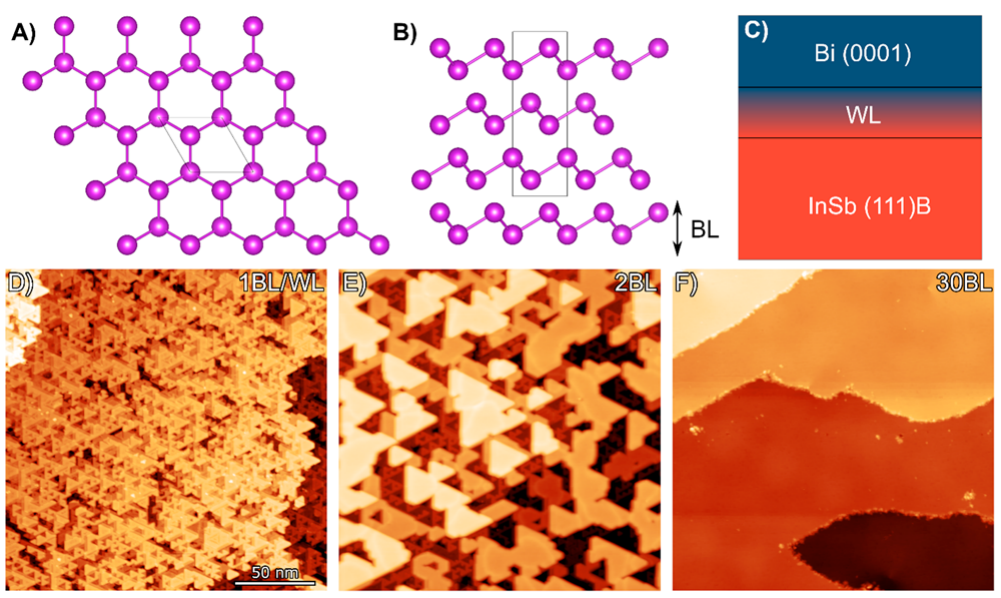
(A) Top view of an individual bilayer (BL) of bismuth, with the unit cell of bismuth outlined. (B) Side view of a bismuth BL, with the unit cell outlined. (C) Layer schematic, consisting of an InSb (111)B substrate, a wetting layer (WL), and the bismuth (0001) thin film. 200 nm × 200 nm STM height images of (D) the bismuth WL on the InSb surface after deposition of 1 BL of bismuth (*I*_T_ = 5 pA, *V*_B_ = 3 V, vertical range = 7.5 Å), (E) a 2 BL thick film, with bismuth islands growing on the wetting layer (*I*_T_ = 5 pA, *V*_B_ = 3 V, vertical range = 16 Å), and (F) a 30 BL thick film, showing a complete film with 1 BL terraces present (*I*_T_ = 5 pA, *V*_B_ = 3 V, vertical range = 15 Å).

Strain soliton formation (also termed ripplocations) in the bismuth thin films is due to the relaxation of the epitaxial tensile strain from the InSb substrate. The strain solitons are local regions of incommensurate bismuth that are relaxed; these solitons separate commensurate (strained) regions of bismuth (Figure 2A). The relaxation of the bismuth is visible in the STM height images of the bismuth thin films as local regions of brighter contrast; the brighter contrast is predominately an out of plane buckling of the thin film due to the soliton relaxation, with little electronic state contribution to the contrast (see Supporting Information S2). In the 2 BL thick film (Figure 2B), soliton lines can be observed to start and terminate at the edges of the bismuth islands. As the bismuth thin film becomes thicker, the soliton morphology can be observed to evolve from predominantly soliton loops in the 3 BL thick film (Figure 2C), to loops and nodes (Figure 2D), and finally to a soliton network (Figure 2E,F). The strain solitons propagate through the different bilayer steps of the bismuth thin film, indicating the strain solitons form at the interface between the bismuth thin film and the wetting layer and propagate throughout the thin film. Upon deposition of the 30 BL of bismuth, no evidence of strain solitons can be observed by STM, and the film appears to have completely relaxed (Figure 2G). See the Supporting Information for XRD supporting this relaxation by forming incommensurate and commensurate regions. Modifications to the local electronic d*I*/d*V* similar to what has been observed at the edges of bismuth crystals reported in ref (16) is observed in the strain solitons (see Supporting Information S3), and further studies are ongoing to understand these states confined to the strain solitons in bismuth. Our results demonstrate that strain solitons can be used to generate localized electronic states at the wafer scale within bismuth thin films, which is potentially enabling scalable future bismuth-based devices that require edge state conduction.

**Figure 2:**
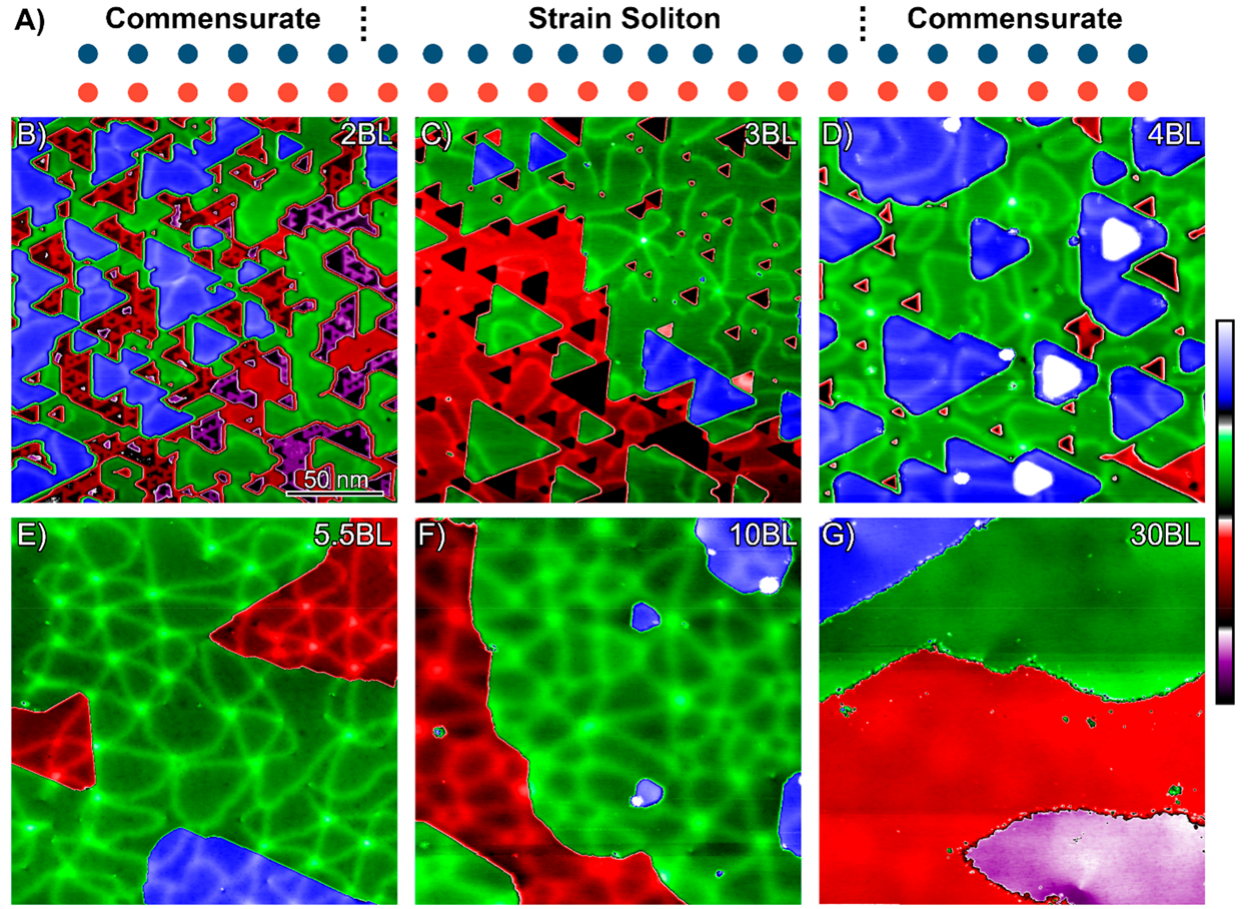
(A) Schematic of a strain soliton acting as the domain wall between commensurate regions of the crystal in the thin film (top layer) on the substrate (bottom layer). 200 × 200 nm STM height images of (B) 2 BL thick film, (C) 3 BL thick film, (D) 4 BL thick film, (E) 5.5 BL thick film, (F) 10 BL thick film, and (G) 30 BL thick film (*I*_T_ = 5 pA, *V*_B_ = 3 V). Vertical scale range: 16 Å.

Analogous to characterization of dislocations, strain solitons can be characterized with the direction of their displacement vector and the direction of propagation.^1^ Tensile strain solitons have a displacement vector perpendicular to the direction of propagation, while shear strain solitons have a displacement vector parallel to the direction of propagation. Tensile and shear solitons have different directions of propagation in the crystal. In materials comprised of honeycomb lattices, tensile strain solitons propagate along the zigzag or <101̅0> direction of the crystal while shear solitons propagate along the armchair or <112̅0> direction of the crystal. Under a biaxial tensile strain, a regularly spaced network of tensile strain solitons is expected.^17,18^ However, irregularly spaced nodes in the soliton networks are observed in the bismuth. Additionally, the bending of the strain solitons is also visible in the STM images for all thicknesses in which strain solitons are visible. As a result of this bending, the strain solitons are of tensile, shear, and mixed types in the bismuth thin film.

To gain further insight into the bending mechanism of the strain solitons, we examine an atomic resolution STM height image of a 5.5 BL thick film where both a large angle bend and a small angle bend in the strain solitons are observed (Figure 3A). Figure 3B and C are enlargements of the large angle and small angle bends to resolve the atoms, respectively. The large angle bend has a soliton bend angle of 150° with the strain soliton converting from a tensile strain soliton to a shear strain soliton, and a point of brighter contrast can be observed at the bend. This point of brighter contrast is an edge dislocation core. The out of plane edge dislocation has an extra half plane in-plane of the bismuth thin film. From the burger circuit, the burger vector is *a*[011̅0], with *a* being the in-plane lattice constant of the bismuth crystal. At a small angle bend of 30°, the strain soliton also converts from a tensile strain soliton to a shear strain soliton. However, at this small angle bend there is no edge dislocation present. The edge dislocations are observed at bends of greater than 30°. We propose that this correlation between the large angle bending of solitons and edge dislocations is due to a locally anisotropic strain field favoring the bending of a strain soliton and the nucleation of an edge dislocation to minimize energy, similar to what has been observed before in uniaxially strained MoSe_2_.^3^

**Figure 3:**
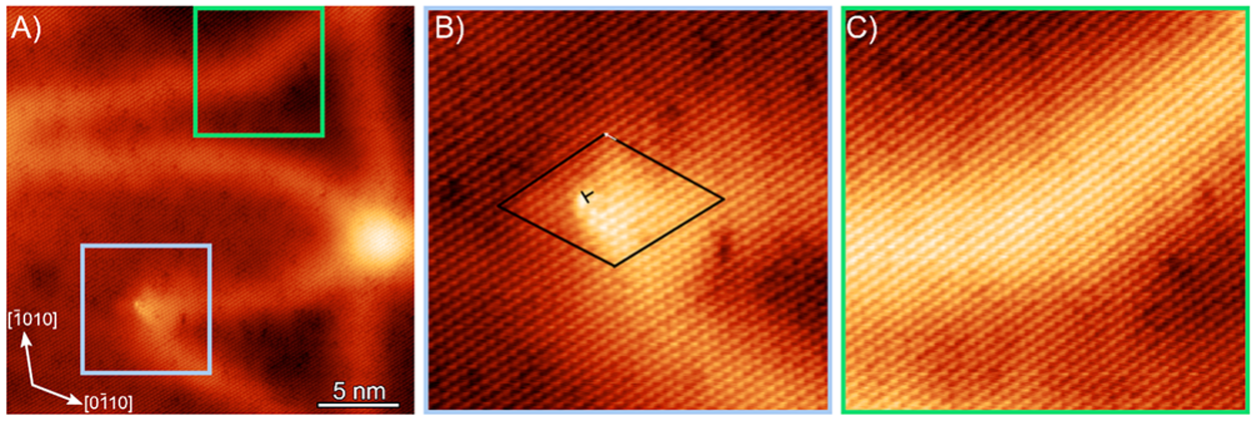
(A) Atomic resolution height image of 5.5 BL thick film (*I*_T_ = 200 pA, *V*_B_ = 0.25 V, vertical range = 2.45 Å). (B) STM height image of a large angle soliton bend, with an edge dislocation present (*I*_T_ = 200 pA, *V*_B_ = 0.25 V, vertical range = 1.33 Å). From the burger circuit, the burger vector is *a*[011̅0]. (C) STM height image of a small angle (30°) bend, no edge dislocations are present at this bend (*I*_T_ = 200 pA, *V*_B_ = 0.25 V, vertical range = 1.12 Å).

The bending and edge dislocation formation is indicative that there is an inhomogeneous strain relaxation. The inhomogeneous strain is likely induced in the thin films, instead of the expected homogeneous, biaxial tensile strain from heteroepitaxial growth. We attribute the local inhomogeneous strain due to interactions between the bismuth and the Sierpiński structure of the wetting layer, observed in Figure 1D, formed from the deposition of the first bilayer of bismuth. Additionally, the change in strain soliton morphology from isolated loops to a soliton network, with the strain solitons becoming predominantly tensile strain solitons as the film thickness increases, is likely due to local interactions with the wetting layer dominating in the thinner films and inducing a locally inhomogeneous strain, which results in the strain soliton morphology favoring bending and loop formation. However, as the film thickness increases, these interactions influence is lessened and the biaxial strain from the substrate favors the formation of a strain soliton network of tensile strain solitons.

Having now studied the bending of the strain solitons, we investigated the strain state of the strain solitons in the bismuth thin film with strain maps from atomic resolution STM images. Figure 4A shows an atomic resolution height image of a 5.5 BL thick film, where a soliton node is observed along with a tensile strain soliton and a shear strain soliton. Figure 4B is an enlargement of the image around the nodes, where the atoms of the bismuth thin film are clearly visible. In the atomic resolution image, only the atoms in the top layer of the bismuth bilayer are imaged, generating the observed hexagonal lattice of atoms. The in-plane strain from the atomic resolution image is determined from the atomic displacements measured with the Lawler-Fujita algorithm,^19^ which is a method of determining atomic displacements similar to geometric phase analysis.^20^ See the Supporting Information in S4 for additional details on the Lawler-Fujita algorithm. The Bragg peaks used for the analysis are circled in the Fourier transform of the atomic resolution image shown in Figure 4C. The in-plane strain maps of the strain solitons and node are shown in Figure 4D–F. The *x* and *y* directions are defined from the image axes, which aligns the *x* direction closely to the [1̅1̅20]. See the Supporting Information for S4 for maps of the principle strain. From the maps, it can be observed that a relaxation of 2.9% ε_*xx*_ tensile strain and 1.3% ε_*xy*_ shear strain is concentrated at the node. Additionally, the tensile strain soliton has the expected relaxation in the direction perpendicular to the direction of propagation with a maximum relaxation of 1.6% ε_*xx*_. Finally, it can be observed that within a shear strain soliton there is 1.4% shear strain present and negligible normal strain present. However, it can also be noted that there is tensile strain relaxation in the direction parallel to the direction of propagation, ε_*yy*_, present in the vicinity of the shear soliton. These results confirm the assignment of strain soliton type based on the direction of soliton propagation. The observed tensile relaxation is consistently larger than 0.8%, which is the expected tensile strain induced by the InSb substrate. This larger than expected relaxation is likely due to the reduction of the lattice constant of bismuth in the ultrathin limit,^21,22^ resulting in larger strains being induced in the thin film when it is strained to InSb for ultrathin films. We observe evidence of the reduction in the lattice constant of relaxed bismuth in our thin films from our reciprocal space maps (see Supporting Information S1). The relaxation of 1.6% tensile strain is similar to the tensile strain of 1.5% required for the formation of strain solitons in uniaxially strained MoSe_2_;^3^ the results suggest that relatively large tensile strains may be required before relaxation by strain solitons can occur.

**Figure 4:**
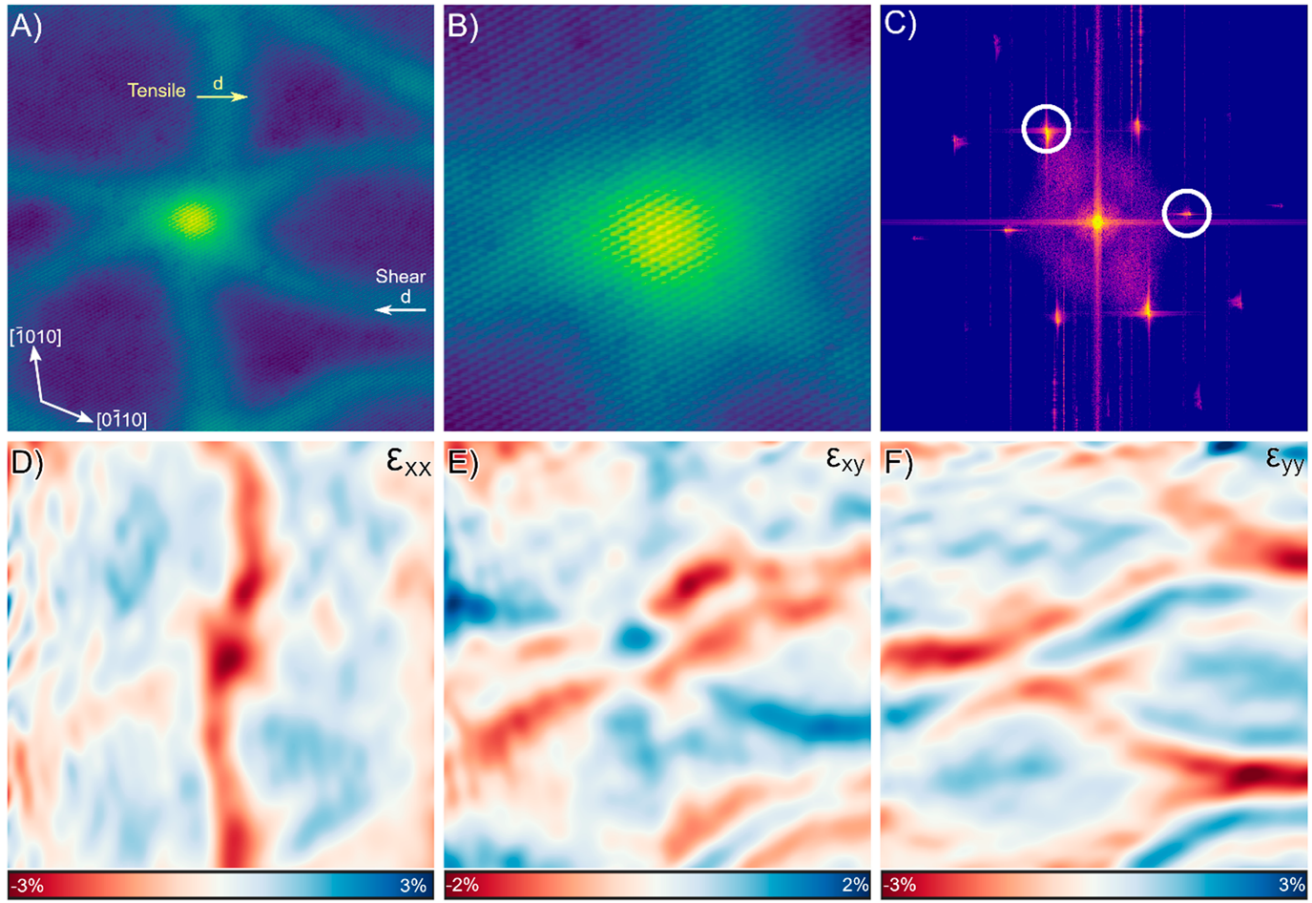
(A) 43.8 × 43.8 nm atomic resolution height image of a soliton node in a 5.5 BL thick film, both a tensile and a shear strain soliton are present with their corresponding displacement vectors indicated (*I*_T_ = 200 pA, *V*_B_ = 0.25 V, vertical range = 2.33 Å). (B) Enlargement of the region near the node, where atoms are visible. (C) FFT of the atomic resolution image; the Bragg peaks used for the strain analysis are circled. Strain maps of the image: (D) ε_*xx*_, (E) ε_*xy*_, and (F) ε_*yy*_ with the *x* and *y* axes defined as the image axes.

In summary, we have demonstrated the formation of strain solitons in a van der Waals-like material with heteroepitaxial growth. The epitaxial strain enables the formation of strain solitons. The bending of strain solitons is observed with large angle bends accompanied by edge dislocations. A change in the strain soliton morphology from loops to a network is observed as the film thickness increases. Normal and shear strain is concentrated in the nodes of the strain solitons. These results indicate that the wetting layer induces an inhomogeneous strain state in the bismuth thin film. Changes in the local electronic states are observed in the strain solitons. Our results demonstrate the importance of interface interactions on the morphology of the strain solitons and enable the formation of strain solitons on a wafer-scale, which is an essential step for scalable and functional devices based on strain solitons.

## Methods

Bi thin films were grown by MBE on unintentionally doped InSb(111)B (Wafer Technology Ltd.) substrates. The substrate surface was cleaned with atomic hydrogen cleaning, see ref (23) for additional details on the surface preparation, which resulted in the 3 × 3 surface reconstruction. Thin films were nucleated at 14 °C followed by low temperature annealing at 80–120 °C for several hours to improve the film morphology. All thicknesses reported in this study are nominal thicknesses based on the calibration of the growth rate from Rutherford backscattering spectrometry.

*In vacuo* STM measurements were performed with an Omicron LT STM instrument at 78 K. Mechanically cut PtIr tips prepared with field emission on Au foil were used for the STM measurements.

## Supporting Information Available

Details of the strain mapping, additional STM images, d*I*/d*V* spectroscopy, and XRD of the thin films (PDF)

## References

[ref1] Alden J. S.; Tsen A. W.; Huang P. Y.; Hovden R.; Brown L.; Park J.; Muller D. A.; McEuen P. L. Strain solitons and topological defects in bilayer graphene. Proc. Natl. Acad. Sci. U.S.A. 2013, 110, 11256–11260. 10.1073/pnas.1309394110. 23798395 PMC3710814

[ref2] Rosenberger M. R.; Chuang H.-J.; Phillips M.; Oleshko V. P.; McCreary K. M.; Sivaram S. V.; Hellberg C. S.; Jonker B. T. Twist Angle-Dependent Atomic Reconstruction and Moiré Patterns in Transition Metal Dichalcogenide Heterostructures. ACS Nano 2020, 14, 4550–4558. 10.1021/acsnano.0c00088. 32167748

[ref3] Edelberg D.; Kumar H.; Shenoy V.; Ochoa H.; Pasupathy A. N. Tunable strain soliton networks confine electrons in van der Waals materials. Nat. Phys. 2020, 16, 1097–1102. 10.1038/s41567-020-0953-2.

[ref4] Kazmierczak N. P.; Van Winkle M.; Ophus C.; Bustillo K. C.; Carr S.; Brown H. G.; Ciston J.; Taniguchi T.; Watanabe K.; Bediako D. K. Strain fields in twisted bilayer graphene. Nat. Mater. 2021, 20, 956–963. 10.1038/s41563-021-00973-w. 33859383

[ref5] Naik M. H.; Jain M. Ultraflatbands and Shear Solitons in Moiré Patterns of Twisted Bilayer Transition Metal Dichalcogenides. PRL 2018, 121, 266401 10.1103/PhysRevLett.121.266401. 30636141

[ref6] Kang D.; Zuo Z.-W.; Wang Z.; Ju W. Multi-shaped strain soliton networks and moiré-potential-modulated band edge states in twisted bilayer SiC. RSC Adv. 2021, 11, 24366–24373. 10.1039/D1RA02139G. 35479044 PMC9036811

[ref7] Ju L.; Shi Z.; Nair N.; Lv Y.; Jin C.; Velasco J. Jr; Ojeda-Aristizabal C.; Bechtel H. A.; Martin M. C.; Zettl A.; Analytis J.; Wang F. Topological valley transport at bilayer graphene domain walls. Nature 2015, 520, 650–655. 10.1038/nature14364. 25901686

[ref8] Yoo H.; Engelke R.; Carr S.; Fang S.; Zhang K.; Cazeaux P.; Sung S. H.; Hovden R.; Tsen A. W.; Taniguchi T.; Watanabe K.; Yi G.-C.; Kim M.; Luskin M.; Tadmor E. B.; Kaxiras E.; Kim P. Atomic and electronic reconstruction at the van der Waals interface in twisted bilayer graphene. Nat. Mater. 2019, 18, 448–453. 10.1038/s41563-019-0346-z. 30988451

[ref9] Jiang L.; Shi Z.; Zeng B.; Wang S.; Kang J.-H.; Joshi T.; Jin C.; Ju L.; Kim J.; Lyu T.; Shen Y.-R.; Crommie M.; Gao H.-J.; Wang F. Soliton-dependent plasmon reflection at bilayer graphene domain walls. Nat. Mater. 2016, 15, 840–844. 10.1038/nmat4653. 27240109

[ref10] Jiang L.; Wang S.; Shi Z.; Jin C.; Utama M. I. B.; Zhao S.; Shen Y.-R.; Gao H.-J.; Zhang G.; Wang F. Manipulation of domain-wall solitons in bi- and trilayer graphene. Nat. Nanotechnol. 2018, 13, 204–208. 10.1038/s41565-017-0042-6. 29358639

[ref11] Ni G. X.; Wang H.; Jiang B.-Y.; Chen L. X.; Du Y.; Sun Z. Y.; Goldflam M. D.; Frenzel A. J.; Xie X. M.; Fogler M. M.; Basov D. N. Soliton superlattices in twisted hexagonal boron nitride. Nat. Commun. 2019, 10, 4360 10.1038/s41467-019-12327-x. 31554808 PMC6761185

[ref12] Tilak N.; Li G.; Taniguchi T.; Watanabe K.; Andrei E. Y. Moiré Potential, Lattice Relaxation, and Layer Polarization in Marginally Twisted MoS2 Bilayers. Nano Lett. 2023, 23, 73–81. 10.1021/acs.nanolett.2c03676. 36576808

[ref13] Schindler F.; Wang Z.; Vergniory M. G.; Cook A. M.; Murani A.; Sengupta S.; Kasumov A. Y.; Deblock R.; Jeon S.; Drozdov I.; Bouchiat H.; Guéron S.; Yazdani A.; Bernevig B. A.; Neupert T. Higher-order topology in bismuth. Nat. Phys. 2018, 14, 918–624. 10.1038/s41567-018-0224-7. 30349581 PMC6195185

[ref14] Liu C.; Zhou Y.; Wang G.; Yin Y.; Li C.; Huang H.; Guan D.; Li Y.; Wang S.; Zheng H.; Liu C.; Han Y.; Evans J. W.; Liu F.; Jia J. Sierpiński Structure and Electronic Topology in Bi Thin Films on InSb(111)B Surfaces. PRL 2021, 126, 176102 10.1103/PhysRevLett.126.176102. 33988396

[ref15] Nagao T.; Sadowski J. T.; Saito M.; Yaginuma S.; Fujikawa Y.; Kogure T.; Ohno T.; Hasegawa Y.; Hasegawa S.; Sakurai T. Nanofilm Allotrope and Phase Transformation of Ultrathin Bi Film on Si(111)–7 × 7. PRL 2004, 93, 105501 10.1103/PhysRevLett.93.105501. 15447414

[ref16] Drozdov I. K.; Alexandradinata A.; Jeon S.; Nadj-Perge S.; Ji H.; Cava R. J.; Andrei Bernevig B.; Yazdani A. One-dimensional topological edge states of bismuth bilayers. Nat. Phys. 2014, 10, 664–669. 10.1038/nphys3048.

[ref17] Lebedeva I. V.; Popov A. M. Commensurate-incommensurate phase transition and a network of domain walls in bilayer graphene with a biaxially stretched layer. PRB 2019, 99, 195448 10.1103/PhysRevB.99.195448.

[ref18] Lebedeva I. V.; Popov A. M. Energetics and Structure of Domain Wall Networks in Minimally Twisted Bilayer Graphene under Strain. J. Phys. Chem. C 2020, 124, 2120–2130. 10.1021/acs.jpcc.9b08306. 32242692

[ref19] Lawler M. J.; Fujita K.; Lee J.; Schmidt A. R.; Kohsaka Y.; Kim C. K.; Eisaki H.; Uchida S.; Davis J. C.; Sethna J. P.; Kim E.-A. Intra-unit-cell electronic nematicity of the high-T_c_ copper-oxide pseudogap states. Nature 2010, 466, 347–351. 10.1038/nature09169. 20631795

[ref20] Hÿtch M. J.; Snoeck E.; Kilaas R. Quantitative measurement of displacement and strain fields from HREM micrographs. Ultramicroscopy 1998, 74, 131–146. 10.1016/S0304-3991(98)00035-7.

[ref21] Lisgarten N. D.; Peppiatt S. J.; Sambles J. R. Lattice parameter changes in thin films of bismuth. J. Phys. C: Solid State Phys. 1974, 7, 2263–2268. 10.1088/0022-3719/7/13/006.

[ref22] Cantele G.; Ninno D. Size-dependent structural and electronic properties of Bi(111) ultrathin nanofilms from first principles. Physical Review Materials 2017, 1, 014002 10.1103/PhysRevMaterials.1.014002.

[ref23] Dong J. T.; Inbar H. S.; Pendharkar M.; van Schijndel T. A. J.; Young E. C.; Dempsey C. P.; Palmstrøm C. J. Electronic structure of InSb (001), (110), and (111)B surfaces. JVST B 2023, 41, 032808 10.1116/6.0002606.

