# Supplementary material for: Strain Solitons in an Epitaxially Strained van der Waals-like Material
Source: Nano Lett. 2024 Mar 18;24(15):4493–7. doi: 10.1021/acs.nanolett.4c00382 (PMC11036392; doi:10.1021/acs.nanolett.4c00382)
Supplement: Supplementary file 1 — nl4c00382_si_001.pdf [file nl4c00382_si_001.pdf]

Supplementary Information for:
Strain Solitons in an Epitaxially Strained van der Waals-like Material

Jason T. Dong[1], Hadass S. Inbar[1], Connor P. Dempsey[2], Aaron N. Engel[1], Christopher J. Palmstrøm[1,2*]

[1]Materials Department, University of California, Santa Barbara, CA 93106

[2]Deparment of Electrical and Computer Engineering, University of California, Santa Barbara, CA 93106

*cjpalm@ucsb.edu

S1: X-Ray Diffraction of Bismuth Thin Films

Lattice parameters, film thickness, and crystallinity information were extracted from high-resolution X-ray diffraction in Figure 1 and confirmed that the films have high crystalline ordering and sharp interfaces. The azimuthal alignment and in-plane strain are further studied, where the epitaxial film-substrate relationship and film strain relaxation are monitored as a function of film thickness. The large-area single-domain orientation observed for Bi (0001)/InSb(111)B is in contrast to the two rotational domains typically seen for Bi films nucleated on Si (111) [1]. Reciprocal space maps (RSMs) of the Bi (1 0 $\bar{1}$ 11) peak for the 5.4 and 13 BL samples are shown in Figure S1(C and D). From the existence of the strained peaks in the RSMs, the film has significant biaxial strain at the ultrathin limit, in agreement with the experimental Poisson ratio of 0.28 for basal plane biaxial strain [2]. The thinner sample has two peaks, a diffused relaxed reflection and a sharp reflection centered at the InSb in-plane lattice constant (with strained lattice parameters: a_S =4.58 Å, c_S =11.81 Å), whereas the thicker 13 BL film has a stronger peak intensity for the relaxed component at a smaller in-plane lattice constant due to more significant relaxation, yet maintains a fraction of a coherently strained film even after strain relaxation occurs (a_s =4.58 Å, c_S =11.86 Å, and for the relaxed peak: a_R =4.524 Å, c_R =11.892 Å).

Interestingly, the relaxed in-plane (a) and trigonal (c) lattice constants shrink/expand (respectively) compared to the literature bulk crystal lattice constants of a=4.546 Å, c =11.862 Å [3]. This contraction in a for thinner films and expansion in c was predicted in computational studies of Bi (111) nanofilms [4,5] and would explain the early onset of strain relaxation observed in our STM scans with tensile strain as high as 3% possible at the 2 BL limit. Therefore, a lattice mismatch higher than the nominal 0.8% tensile strain (with respect to the Bi bulk lattice constant) is applied at the ultrathin limit when Bi is nucleated on InSb.

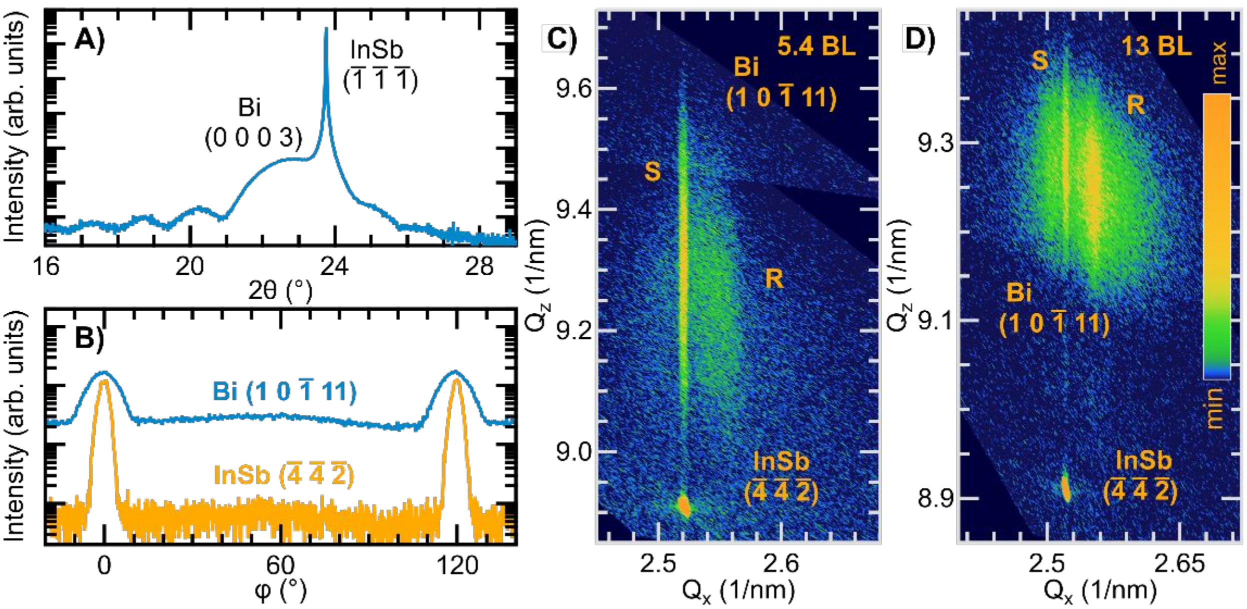

Figure 1: Epitaxy of bismuth on InSb (111)B. (A) θ-2θ XRD scan exhibiting thickness fringes indicating a sharp interface. (B) In-plane φ scan of the Bi (1 0 $\bar{1}$11) and InSb ($\bar{4}\,\bar{4}\,\bar{2}$) reflections showing the 3-fold symmetry of a single crystal domain orientation. Reciprocal space map of (C) 5.4 BL and a (D) 13 BL thick film showing partial relaxation, with both coherently strained (S) and relaxed peaks (R) present.

S2: Additional STM Images

Additional STM images at different voltages (+3 V and -2V) at two different locations for a 10 BL thick film are shown in Figure 2. The soliton heights are approximately 0.4 Å in both images, and the soliton morphology appears to be the same. This result is indicative that the brights regions of contrast in the STM images are predominately structural in origin and there is little contribution due to local density of states differences in the contrast of the images. The brighter regions of contrast could be potentially due to an out of plane buckling in the film in the soliton due to the strain relaxation.

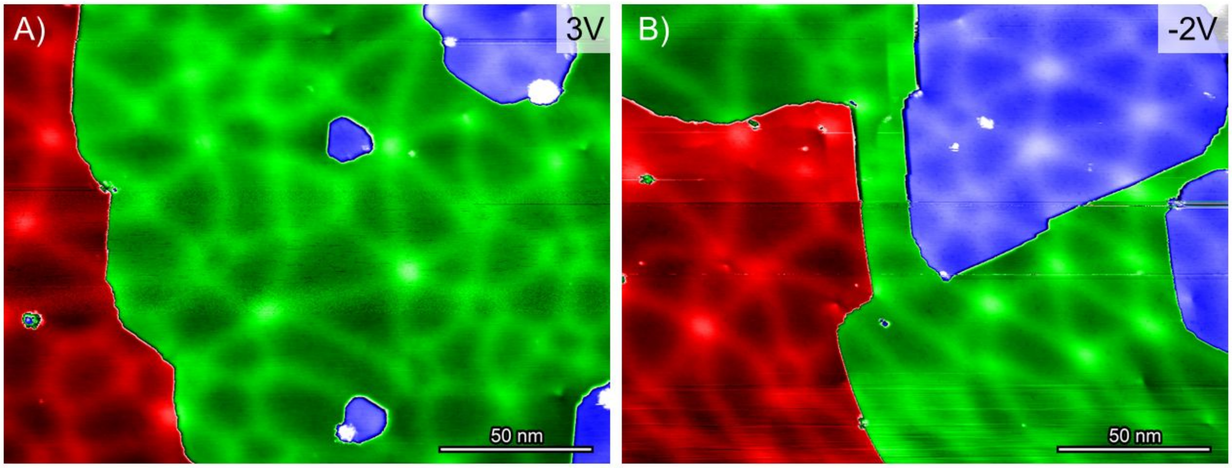

Figure 2: STM height images of 10 BL thick bismuth film at A) $V_B = 3$ V, $I_T = 5$ pA, vertical range = 10.2 Å and B) $V_B = -2$ V, $I_T = 5$ pA, vertical range = 10.7 Å some piezo creep is visible in bottom of the image. The images were taken at different locations on the film.

S3: dI/dV Spectroscopy

dI/dV spectroscopy of the 5.5 BL thick bismuth thin film on the center of a terrace and on the edge of a terrace in Fig. 3. The region studied is shown in Fig. 3A. Significant changes to the dI/dV spectra occur when the measurement is performed at the edge. A similar shift in the peak near 0.2 V towards 0 V was observed as the peak shift observed due to one dimensional topological edge states on bismuth bulk crystals [6]. We also observe additional changes in some features at 0 V and at voltages further from the Fermi-level. Work is ongoing to understand these changes.

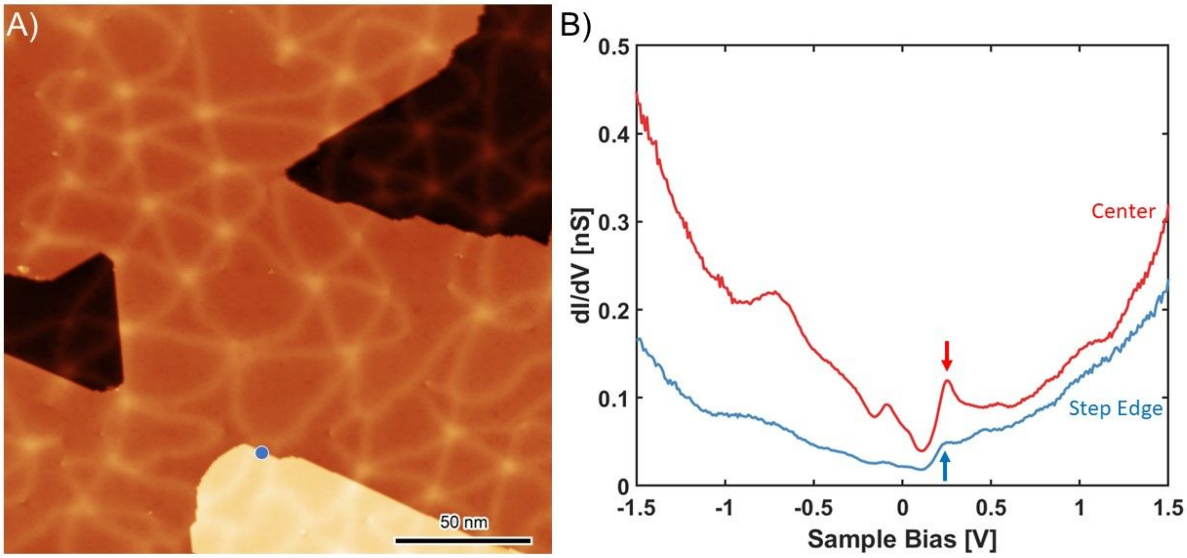

Figure 3: STM height images of 5.5 BL thick bismuth film at A) $V_B = 3$ V, $I_T = 5$ pA, vertical range = 10.7 Å and B) STS in middle of region between strain solitons and on the strain soliton node.

dI/dV spectroscopy of the 5.5 BL thick bismuth thin film on the strain soliton node is shown Fig. 4. The location of the spectra take are indicated by the dots in Fig. 4A. Significant changes to the dI/dV spectra occur when the measurement is performed on the strain soliton node. Similar to what was observed in the 1D topological edge states in bulk bismuth crystals [6], a small shift in the peak near 0.2 V towards 0V is observed in the strain soliton node. Additionally, a large peak shift occurs near -0.75 V towards more negative voltages is also observed in the strain soliton node. Further investigations are ongoing to understand the changes in the electronic states in the strain solitons.

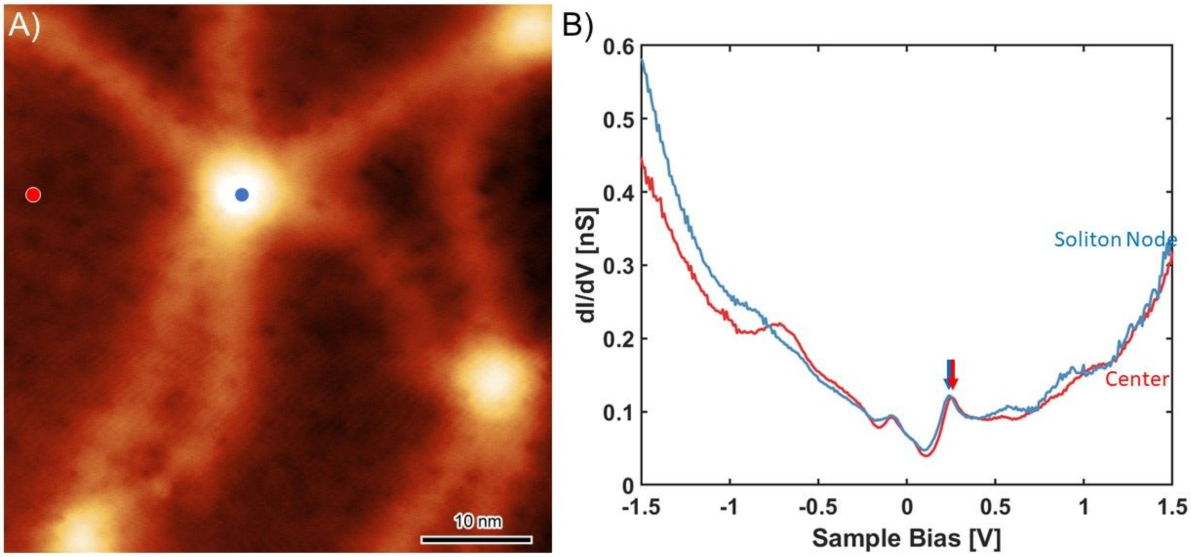

Figure 4: STM height images of 5.5 BL thick bismuth film at A) $V_B = 0.2$ V, $I_T = 500$ pA, vertical range = 2.04 Å and B) STS in middle of region between strain solitons and on the strain soliton node.

S4: Strain Mapping

Atomic displacements were measured using the Lawler-Fujita algorithm [7]. In addition to displacements due strain, displacements of the atoms measured by STM can be due to piezo nonlinearity, thermal drifts, and piezo hysteresis. To remove these artifacts, a low order polynomial is subtracted from the displacement matrices [8]. Following this subtraction, the 2D strain components are calculated using the derivatives of the displacement matrices [9].

The principle strain represent the maximum and minimum strain experienced by the material, and is way of representing strain without a coordinate system [10]. The measured strain can be converted to the principle strain with the following equations:

$$\epsilon_{max} = \frac{\epsilon_{xx} + \epsilon_{yy}}{2} + \sqrt{\left(\frac{\epsilon_{xx} - \epsilon_{yy}}{2}\right)^2 + (\epsilon_{xy})^2}$$

$$\epsilon_{min} = \frac{\epsilon_{xx} + \epsilon_{yy}}{2} - \sqrt{\left(\frac{\epsilon_{xx} - \epsilon_{yy}}{2}\right)^2 + (\epsilon_{xy})^2}$$

The maximum strain represents the most positive strain experienced by the material, and the minimum strain represents the most negative strain by the material. With these strains the maximum shear strain experienced by the material can also be calculated using the following equation:

$$\gamma_{max} = \epsilon_{max} - \epsilon_{min}$$

Maps of the principle strain and the maximum shear strain are shown along with the atomic resolution image in Fig. 5. We can observe that the strain solitons cause relaxation of the bismuth thin film, and shear strain is present with the strain solitons. All of the soliton types have shear strain present within the material, due to the solitons not inducing hydrostatic relaxation of the material and relaxing the material locally typically in a single direction, and shear strain is always required in order to allow for shape of the material to change to allow for the uniaxial relaxation.

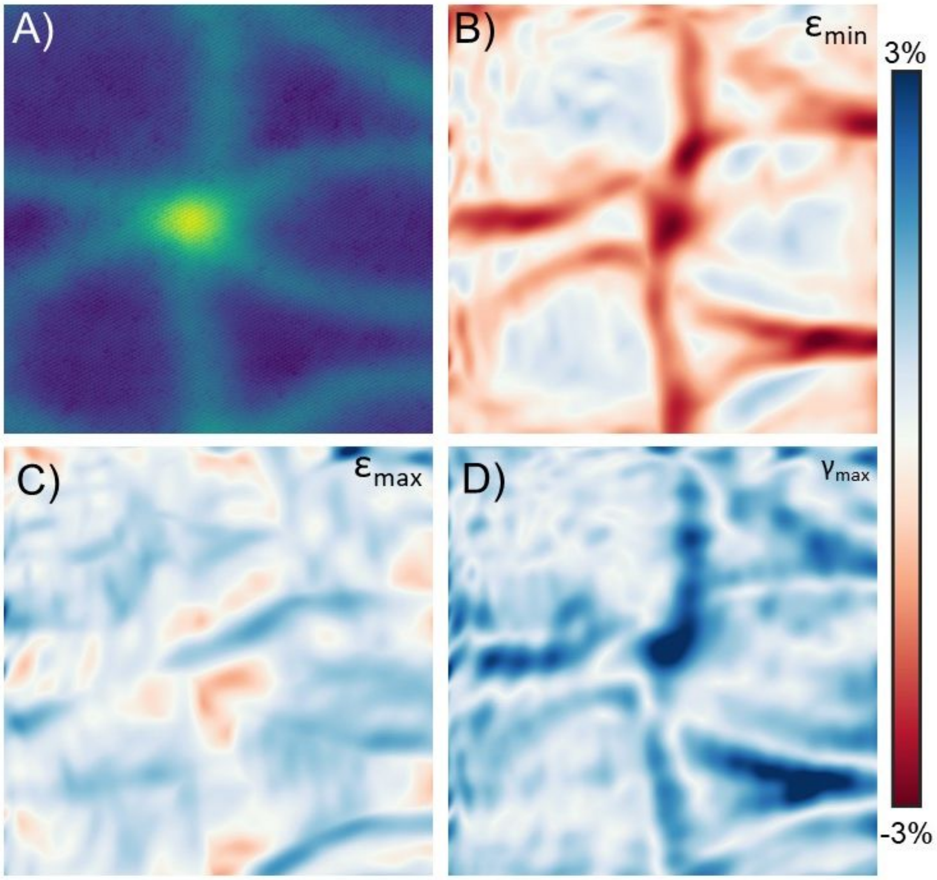

Figure 5: Atomic resolution STM height image of strain soliton node for 5.5 BL thick film (I_T = 200 pA, V_B = 0.25 V, vertical range = 2.33 Å).

References

[1] Shirasawa T.; Ohyama, M.; Voegeli, W.; Takahashi, T.; *Phys. Rev. B* **2011**, 84, 075411. DOI: 10.1103/PhysRevB.84.075411

[2] Eckstein, Y.; Lawson, A. W. ; Reneker, D. H.; *J. Appl. Phys.* **1960**, 31, 1534. DOI: 10.1063/1.1735888

[3] Mönig, H.; Sun, J.; Koroteev, Y. M.; Bihlmayer, G.; Wells, J.; Chulkov, E. V.; Pohl, K.; Hofmann, P.; *Phys. Rev. B* **2005**, 72, 085410. DOI: 10.1103/PhysRevB.72.085410
[4] Cantele, G.; Ninno, D.; *Phys. Rev. Mater.* **2017**, 1, 014002. DOI: 10.1103/PhysRevMaterials.1.014002
[5] Inbar, H. S.; Zubair, M.; Dong, J. T.; Engel, A. N.; Dempsey, C. P., Chang, Y. H.; Nishihaya, S.; Khalid, S.; Fedorov, A. V.; Janotti, A.; Palmstrøm, C. J.; *arXiv*, **2023**, 2302.00803 DOI: 10.48550/arXiv.2302.00803
[6] Drozdov, I. K.; Alexandradinata, A.; Jeon, S.; Nadj-Perge, S.; Ji, H.; Cava, R. J.; Bernevig, B. A.; Yazdani, A.; *Nature Physics* **2014**, 10, 664-669. DOI: 10.1038/NPHYS3048
[7] Lawler, M. J.; Fujita, K.; Lee, J.; Schmidt, A. R.; Kohsaka, Y.; Kim, C. K.; Eisaki, H.; Uchida, S.; Davis, J. C.; Sethna, J. P.; Kim, E.-A.; *Nature* **2010**, *466*, 347-351. DOI: 10.1038/nature09169
[8] Zeljkovic, I.; Walkup, D.; Assaf, B. A.; Scipioni, K. L.; Sankar, R.; Chou, F.; Madhavan, V.; *Nat. Nanotechnol* **2015**, *10*, 849-853. DOI: 10.1038/nnano.2015.177
[9] Hÿtch, M.J.; Snoeck, E.; Kilaas, R.; *Ultramicroscopy* **1998**, 74, 131-146. DOI: 10.1016/S0304-3991(98)00035-7
[10] Kazmierczak, N. P.; Van Winkle, M.; Ophus, C.; Bustillo, K. C.; Carr, S.; Brown, H. G.; Ciston, J.; Taniguchi, T.; Watanabe, K.; Bediako, D. K.; Strain fields in twisted bilayer graphene. *Nature Materials* **2021**, *20*, 956-963. DOI: 10.1038/s41563-021-00973-w
